# Effects of Cannabidiol, Hypothermia, and Their Combination in Newborn Rats with Hypoxic-Ischemic Encephalopathy

**DOI:** 10.1523/ENEURO.0417-22.2023

**Published:** 2023-05-04

**Authors:** Francisco J. Alvarez, Antonia A. Alvarez, José. J. Rodríguez, Hector Lafuente, M. Josune Canduela, William Hind, José L. Blanco-Bruned, Daniel Alonso-Alconada, Enrique Hilario

**Affiliations:** 1 Biocruces Bizkaia Health Research Institute, 48903 Barakaldo, Spain; 2 Department of Cell Biology, University of the Basque Country, 48940 Leioa, Spain; 3 Functional Neuroanatomy Group, Biocruces Health Research Institute, 48903 Barakaldo, Spain; 4 Basque Foundation for Science (IKERBASQUE), 48009 Bilbao, Spain; 5 Department of Neurosciences, Medical Faculty, University of the Basque Country (UPV/EHU), 48940 Leioa, Spain; 6 Biodonostia Health Research Institute, 20014 Donostia, Spain; 7 Department of Neurosciences, University of the Basque Country, 48940 Leioa, Spain; 8 Jazz Pharmaceuticals, Cambridge CB24 9BZ, United Kingdom; 9 Department of Pediatric Surgery, Cruces University Hospital, OSI-Ezkerraldea Enkarterri Cruces, 48903 Barakaldo, Spain

**Keywords:** cannabidiol, hypothermia, hypoxic-ischemic encephalopathy, neuroprotection, neonate, juvenile

## Abstract

Therapeutic hypothermia is well established as a standard treatment for infants with hypoxic-ischemic (HI) encephalopathy but it is only partially effective. The potential for combination treatments to augment hypothermic neuroprotection has major relevance. Our aim was to assess the effects of treating newborn rats following HI injury with cannabidiol (CBD) at 0.1 or 1 mg/kg, i.p., in normothermic (37.5°C) and hypothermic (32.0°C) conditions, from 7 d of age (neonatal phase) to 37 d of age (juvenile phase). Placebo or CBD was administered at 0.5, 24, and 48 h after HI injury. Two sensorimotor (rotarod and cylinder rearing) and two cognitive (novel object recognition and T-maze) tests were conducted 30 d after HI. The extent of brain damage was determined by magnetic resonance imaging, histologic evaluation, magnetic resonance spectroscopy, amplitude-integrated electroencephalography, and Western blotting. At 37 d, the HI insult produced impairments in all neurobehavioral scores (cognitive and sensorimotor tests), brain activity (electroencephalography), neuropathological score (temporoparietal cortexes and CA1 layer of hippocampus), lesion volume, magnetic resonance biomarkers of brain injury (metabolic dysfunction, excitotoxicity, neural damage, and mitochondrial impairment), oxidative stress, and inflammation (TNFα). We observed that CBD or hypothermia (to a lesser extent than CBD) alone improved cognitive and motor functions, as well as brain activity. When used together, CBD and hypothermia ameliorated brain excitotoxicity, oxidative stress, and inflammation, reduced brain infarct volume, lessened the extent of histologic damage, and demonstrated additivity in some parameters. Thus, coadministration of CBD and hypothermia could complement each other in their specific mechanisms to provide neuroprotection.

## Significance Statement

Cannabidiol and hypothermia act on some common processes related to hypoxic-ischemic brain damage, modulating excitotoxicity, inflammation, and oxidative stress. The two therapies in combination do not compete against each other in modulating these processes, but rather produce additive neuroprotective effects. Furthermore, in the instances where there was not an additive effect, the combination of cannabinoid with hypothermia often resulted in a significantly superior profile compared with hypothermia alone, being a promising observation for the clinic. These results justify interest in cannabidiol for developing a combined treatment with hypothermia to increase the number of hypoxic-ischemic infants that benefit from treatment.

## Introduction

Management of newborn hypoxic-ischemic encephalopathy (HIE) is determined by its complex pathophysiology, and any neuroprotective strategy must act on several factors, mainly excitotoxicity, oxidative stress, and inflammation (Drury et al., 2014). Therapeutic hypothermia (HT) demonstrated these properties, being the only approved therapy with neuroprotective efficacy in human newborns (Edwards et al., 2010; Jacobs et al., 2013; Heursen et al., 2017). However, these benefits are partial (Jacobs et al., 2013), and the evidence from 11 randomized controlled trials (>1500 infants) showed that therapeutic hypothermia provided some benefit to newborns with moderate HIE, but hypothermia was ineffective in infants with severe encephalopathy (Edwards et al., 2010; Heursen et al., 2017). Although there is considerable evidence that some infants with moderate encephalopathy can benefit overall from therapeutic hypothermia, current clinical practice means that no routine treatment is effective in severe cases. On this basis, current investigation is focused on combination therapies with hypothermia (Hobbs et al., 2008; Lafuente et al., 2016; Barata et al., 2019).

Cannabidiol (CBD) has been shown to reduce brain damage in experimental neonatal HIE models (Alvarez et al., 2008; Castillo et al., 2010; Lafuente et al., 2011; Pazos et al., 2012, 2013). Some of the properties associated with the use of CBD, which may underlie these effects in models of HIE, include, among others, antioxidant and anti-inflammatory properties, and reductions of Ca^2+^ influx and glutamate release, but a direct effect of CBD on these parameters has not been proven yet (Alvarez et al., 2008; Castillo et al., 2010; Lafuente et al., 2011; Pazos et al., 2012, 2013). Also, the neuroprotective action of CBD was not associated with significant side effects in these animal models, and extracerebral additional benefits were associated with cardiac, hemodynamic, and ventilatory effects (Alvarez et al., 2008).

After HI insult, neonatal neuroprotection induced by CBD has been demonstrated in different animal models: 1 mg/kg, s.c., in neonatal mice (Mohammed et al., 2017), 1 mg/kg, s.c., in neonatal rats (Pazos et al., 2012), and 0.1 or 1 mg/kg, i.v., in piglets (Alvarez et al., 2008; Lafuente et al., 2011, 2016; Pazos et al., 2013; Garberget al., 2016). However, higher doses from 5 to 50 mg/kg, i.v., in global HI piglets did not offer neuroprotection in the first hours after severe global hypoxia-ischemia (Garberg et al., 2017), but, moreover, a positive correlation between the degree of hypotension and the plasma concentrations of CBD was observed at these high doses (Garberg et al., 2017). In contrast, a well tolerated dose of CBD 1 mg/kg, i.v., did not produce unwanted cardiovascular effects in HI piglets (Pazos et al., 2013; Garberg et al., 2016; Lafuente et al., 2016). Taken as a whole, these studies support the neuroprotective role of CBD at low doses (<5 mg/kg) as a pharmacological agent for newborns with HIE.

However, there are limited data on how CBD might work together with hypothermia. Two studies demonstrated that combining 1 mg/kg, i.v., CBD with hypothermia in short-term studies (6 or 72 h) was safe and provided neuroprotection in HI piglets (Lafuente et al., 2016; Barata et al., 2019). The combined effect of hypothermia and CBD to reduce excitotoxicity, inflammation, and oxidative stress, as well as cell damage, was greater than either that of hypothermia or CBD alone, indicating additivity. However, before considering the combination of both therapies for clinical use, it is important to test this in more than one species and to determine whether these adjuvant therapies remain effective throughout the secondary deterioration period, which will require additional experiments extending the monitoring neonatal period beyond 72 h after hypoxia-ischemia, at least to a juvenile phase (Juul and Ferriero, 2014). The current study is the first one to investigate the effects of combining CBD (two doses) with HT in neonatal rats (7 d old) and on long-term outcomes in juvenile rats (37 d old).

## Materials and Methods

### Ethical approval

The experimental protocol satisfies European and Spanish regulations for the protection of experimental animals (86/609/EEC and RD 53/2013). The study protocol was evaluated by the Animal Welfare Body from the University of the Basque Country and was performed in its experimental surgical theatres (permit no. M20/2015/055).

All experimental procedures were designed and conducted by personnel qualified in Laboratory Animal Science, following the Federation of European Laboratory Animal Science Associations (FELASA) recommendations on categories B and C. All surgery was performed under adequate anesthesia and analgesia, and all efforts were made to minimize suffering and to reduce the number of animals used. Also, all experimental procedures on animal welfare (anesthesia and analgesia, drug and substance administration) and the humane killing of the animals were conducted in compliance with FELASA recommendations.

### Animals

Twenty-five pregnant-specific pathogen-free female Wistar rats (Harlan) of identical age (8 weeks; weight, 149–191 g) at the start of the study were used. Seven days after birth [postnatal day 7 (P7)], rat pups were used for the experimental procedure (*N* = 262; Table 1). At P21, male and female rats were separated into small single-sex social groups (6 rats/cage) to avoid space restriction and inbreeding.

**Table 1:** Total number of animal used for pharmacokinetic and pharmacodynamic studies as well as sex assignment (male/female) per group^*a*^

	P7 animals assigned to PKS^*b*^ total, *n* (male/female)	P14 animals assigned to NS and NIS total, *n* (male/female)	P37 animals assigned to NFS, NS, NIS, and BS total, *n* (male/female)
SHAM	0 (0/0)	10 (6/4)	10 (6/4)
VEH-NT	0 (0/0)	10 (4/6)	10 (7†/4)
VEH-HT	0 (0/0)	10 (6/4)	10 (6†/5)
CBD 0.1-NT	30 (14/16)	10 (5/5)	10 (6/4)
CBD 0.1-HT	30 (16/14)	10 (6/4)	10 (4/6)
CBD 1-NT	30 (17/13)	10 (5/5)	10 (5/5)
CBD 1-HT	30 (16/14)	10 (5/5)	10 (6/4)
Total	120 (63/57)	70 (37/33)	70 (38/32)

PKS, PK studies; NS, neuropathological studies; NIS, neural injury studies; NFS, neurologic functional studies; BS, biochemical studies.

a One animal excluded by sudden unexpected death.

b Five additional animals used as a reference for analytical purposes are not included in the table.

Animals were housed at a constant temperature of 22 ± 1°C, relative humidity of 60 ± 5%, and under a 12 h light/dark cycle with the lights turned on at 8:00 A.M. Food and water was supplied *ad libitum*, and no differences were observed in the amount of standard food eaten by the different groups of rats during the study period. Control welfare-related assessments were conducted daily during each trial. All animals were weighed and rectal temperature determined once a week.

### Experimental protocol

The protocol was based on the model developed by Vannucci’s group (1980), which has been extensively described previously (Fernández-López et al., 2007). Briefly, P7 is an age that is representative of a human infant born at preterm or near term (32–36 weeks) and has commonly been used in NHIE studies in the literature, so P7 rat pups were used in all experiments; however, since 2015 it has now been shown that P10 is the most appropriate age at which to use rat pups (Patel et al., 2015). Only litters with 11 or 12 pups were used to obtain 10 animals for random treat assignment as well as 1 or 2 sentinel pups for temperature control (Dubovický et al., 2008; Chahoud and Paumgartten, 2009). The pups were weighed on the day of the experiment to determine the correct dose of drug treatment.

Pups were anesthetized by inhaled isoflurane (5% induction, 1.5% maintenance). The neck skin of the animal was cleaned with 0.5% chlorhexidine before surgery, which was conducted in aseptic conditions. The left common carotid artery was exposed, carefully isolated from surrounding tissues, and cut by electrocautery (Low Temperature Micro Fine Tip 454 Cautery Pen, Stoelting). Silk 3–0 sutures were used to close the skin wound. After recovery from anesthesia, pups were returned to their dams for 3-4 h.

Pups were placed into 1000 ml airtight glass containers (five animals each) in a water bath at 36.5°C, and then exposed to 10% O_2_ + 90% N_2_ (hypoxic atmosphere) for 120 min. After the HI insult, pups were resuscitated when needed by cardiorespiratory stimulation for a maximum of 10 min. Pups not resuscitating within 10 min were not included in the study (total number entering procedure, 260; number of decedents, 2). Although treatment with 10% O_2_ could induce a mild insult compared with what is reported in the literature, increased hypoxic interval, from 75 or 90 min to 120 min, are related to a more severe injury (Fernández-López et al., 2007; Pazos et al., 2012).

Rat pups were randomly assigned to be maintained in individual chambers in an incubator at 37.5°C [normothermia (NT)] or 32.0°C (HT), based on previous studies (Thoresen et al., 1996; Wagner et al., 2002). Moreover, the “normothermic” temperature of 37.5°C measured rectally was selected by corresponding to the actual clinical practice in human newborns.

Pups were maintained under HT conditions for 48 h and rewarmed for 12 h (at ∼0.5°C/h). Core temperatures were recorded in each chamber in “sentinel pups” (“probe animals”) carrying a rectal temperature probe to monitor the temperature during the whole experimental period, and they were later excluded (K thermistor, Xindar; probe diameter, <1 mm) during previously related phases (HT treatment for 48 h and the rewarming phase for 12 h). Rectal temperature was maintained within ±0.2°C of the target using a servo-controlled water bath (Precisdig, JP Selecta) inside the chamber. In P7 rats, rectal temperature correlates within 0.1°C with brain temperature (Thoresen et al., 1996). In all animals, rectal temperature was measured during a few seconds every 12 h during the first 3 d.

Similarly, NT pups were maintained in individualized cages for similar time periods (48 h). All pups were fed every 6 h with a puppy milk replacer (10 ml/kg; Esbilac, PetAg) by orogastric gavage (20 ga plastic feeding tubes, Instech Lab). Then, all pup rats were returned to their dams and weaned at P21, as above described.

### Treatment

Thirty minutes after HI insult, rat pups were first randomized by sealed envelope to normothermia or hypothermia treatment. Then, HI-injured pups treated with NT or HT were again randomized for drug administration by sealed envelope. All neonatal rats received placebo solution, 0.1 or 1 mg/kg CBD (GW Research, now part of Jazz Pharmaceuticals) by intraperitoneal injection, 30 min, 24 h, and 48 h after insult. Brain damage and neuroprotective effects were assessed by histologic, neuroimaging, biochemical, and neurobehavioral studies. A sham-operated group of animals without hypoxia-ischemia or drug treatment was included as reference.

For the pharmacokinetic (PK) analysis of CBD in normothermia and hypothermia, rats received CBD 0.1 or 1 mg/kg, i.p., 30 min after hypoxia-ischemia. Pups were humanely killed by decapitation at 0.5, 1, 6, 12, 24, or 36 h after initial drug administration. Brain and plasma samples were collected and immediately frozen to determine drug concentration by liquid chromatograph with liquid chromatography-tandem mass spectrometry (analysis conducted at LGC). Brains from five animals without HI insult or drug treatment were used as reference for analytical purposes.

### Neurologic functional tests

All animals were acclimatized to the test room before trials during previous days. P37 animals underwent two motor and two cognitive tests, with 1 h latency between two consecutive tests.

The rotarod test assesses locomotor deficit in neurodegenerative disease models in rodents. The accelerating protocol provides a more discriminative test to correlate locomotor deficits against lesion size (Monville et al., 2006). Rats were placed on a cylinder with linear acceleration from 4 to 40 rpm for 5 min. The time until falling down was recorded. Motor coordination was assessed by comparing the latency to fall in seconds on the first trial between treatment groups.

The cylinder rearing test (CRT) examines lateral bias of sensorimotor deficits (Grow et al., 2003). Rats were placed into a glass cylinder for 5 min, and the number of times the left and/or the right forepaw was placed on the cylinder inner surface was recorded. Right deficit (left preference) was assessed as follows: ([no. left forepaw placements] – [no. right forepaw placements])/(no. left, right, and both forepaw placements) × 100.

The novel object recognition test explores nonspatial working memory (Dere et al., 2007; Broadbent et al., 2010). Rats were exposed to two identical objects for 5 min. One hour later, one of the objects was replaced for a new one (different in shape, color, and size), and all animals were recorded for 5 min. The time spent by each animal to explore the novel and the familiar object was used to calculate the discrimination index (DI). DI uses the difference in exploration time for familiar objects (TF) and time for novel objects (TN), divided by the total amount of exploration of both types of objects, as follows: DI = (TN – TF)/(TN + TF) (Dere et al., 2007). This result can vary between +1 and −1, where a positive score indicates more time spent with the novel object, a negative score indicates more time spent with the familiar object, and a zero score indicates a null preference (Antunes and Biala, 2012).

Spontaneous alternation in a T-maze is used to study spatial learning and memory (Deacon and Rawlins, 2006). The full experiment consisted of the following three parts during 3 consecutive days: habituation, training, and testing. Two hours before the training and testing phases, daily food rationing was performed and replaced by 50 mg of goodie rewards (Fruit Crunchies, Bioserv) as indicated by protocol (Deacon and Rawlins, 2006). The data obtained from the T-maze consists of the number of correct versus incorrect arm entries in each trial (at least a first free run and five runs per animal), for no longer than 15 min. The percentages of correct arm choices were graphed and compared across control and treated disease groups (Deacon and Rawlins, 2006).

### Amplitude-integrated electroencephalography

Brain activity was monitored using a two-channel bed electroencephalography (EEG) monitor (EEG-SMT, Olimex) with five needle electrodes placed in frontal and posterior biparietal placements for four recording electrodes and one reference electrodes (Tucker et al., 2009). The “raw” EEG signal was performed at 100 Hz sampling frequency and in the frequency domain from 0 to 60 Hz (low-pass filter, 0.2 Hz; high-pass filter, 59 Hz; Zayachkivskyet al., 2013). The raw EEG signal was registered only at P37, under general anesthesia with isoflurane, as described above, for a duration of 30 min. The amplitude integrated EEG signal was obtained via a step-by-step signal-processing method (Zhang et al., 2011). Six steps were applied to calculate a compact amplitude-integrated EEG (aEEG) tracing and the upper/lower margin using raw EEG data: (1) asymmetrical data filtering (to provide equal weight to the energy of nonrhythmic components at each frequency); (2) absolute value evaluation (to acquire the amplitude information of EEG data with a biphasic nature); (3) envelope detection (to monitor the brain function by displaying the amplitude trend of brain activity); (4) tracing compression (to obtain a bird’s-eye-view of the cerebral function over a long duration); (5) segmentation and terminal point extraction (to obtain a compact aEEG, simplifying the full tracing into a series of vertical lines); and (6) margin calculation (to obtain a smooth and representative margin, the median amplitude of every successive 20 terminal points was used). Furthermore, raw EEG traces were manually reviewed for the presence of seizures, which are described as periods of a sudden increase in voltage, accompanied by a narrowing of the band of aEEG activity, and followed by a brief period of suppression (Alvarez et al., 2008).

### Brain samples

Animals were killed by decapitation at P14 and P37. The brains of the animals were immediately removed from the skull, and the brains of half of the animals in each group were frozen with liquid nitrogen (<1 min), while the brains of the other half were fixed by immersion in paraformaldehyde 4%. All brains were stored individually. The paraformaldehyde-fixed brains (*N* = 5/group at P14 and P37) were used for histologic studies, and the frozen brains (*N* = 5/group at P14 and P37) were used for both magnetic resonance imaging (MRI) and proton magnetic resonance spectroscopy (H^+^-MRS).

### Histologic studies

The brains were embedded in paraffin, and coronal sections (4 μm) were cut and mounted on glass slides for hematoxylin-eosin and Nissl staining. Three consecutive sections corresponding to coordinates in the rat brain atlas (from bregma, −4.44; interaural, 4.56; Paxinos and Watson, 2007, their Figure 7*0*) were selected for analysis by an examiner blinded to the experimental group of the animal. The degree of brain damage (neuropathological score) in the CA1 area of the ipsilateral hippocampus and the ipsilateral temporoparietal cortex were scored as follows (Mohammed et al., 2017; Barata et al., 2019): 0 = normal; 1 = few neurons damaged (1–5%); 2 = several neurons damaged (6–25%); 3 = moderate number of neurons damaged (26–50%); 4 = more than half of neurons damaged (51–75%); and, 5 = majority of neurons damaged (>75%) or absent hippocampus. The mean of three sections from each animal was determined. The temporoparietal cortex includes the following areas: parietal cortex, posterior area, dorsal part; parietal cortex, posterior area, rostral part; primary somatosensory cortex; primary auditory cortex; secondary auditory cortex, dorsal area; and secondary auditory cortex, ventral area.

### Proton magnetic resonance spectroscopy

Ipsilateral samples (temporoparietal cortex and hippocampus) from frozen brains of the seven groups (*N* = 5 animals/group) at P14 and P37 were analyzed in the Biomedical Research Institute Alberto Sols (Autónoma University, Madrid) as previously described (Pazos et al., 2012). H^+^-MRS was performed at 500.13 MHz using an 11.7 T spectrometer (AMX500, Bruker) operating at 4°C on frozen samples (3 mg) placed within a 50-l zirconium oxide rotor with a cylindrical insert and spun at 4000 Hz. All spectra were processed using TOPSPIN software, version 1.3 (Bruker). Spectra were phased, baseline corrected, and referenced to the sodium (3-trimethylsilyl)−2,2,3,3-tetradeuteriopropionate singlet at δ 0 ppm. Lactate/*N*-acetylaspartate (Lac/NAA; metabolic dysfunction), glutamate/NAA (Glu/NAA; excitotoxicity), NAA/choline (NAA/Cho; neural damage), and lactate/creatine (Lac/Cr; mitochondrial impairment) ratios were calculated.

### Magnetic resonance imaging

Whole fixed brains of the seven groups (*N* = 5 animals/group) at P14 and P37 were transferred to the Biomedical Research Institute Alberto Sols (Autónoma University, Madrid), where an MRI scan was performed on an MRI (BioSpec BMT 47/40, Bruker-Medical) operating at 4.7 T, equipped with an avoid actively shielded gradient insert with 11.2 cm bore, a maximal gradient strength of 200 mT/m and 80 μs rise time, and a home-made 4 cm surface coil, as previously described (Pazos et al., 2012). T2-weighted images (T2WIs) were acquired with a multislice rapid acquisition (TR = 3.4 s; rapid acquisition with relaxation enhancement factor = 8; interecho interval = 30 ms; effective TE = 120 ms; matrix size, 256 × 256; pixel dimensions, 117 × 117 μm; FOV = 3 cm^2^).The slice package consisted of 13 consecutive slices of 1.0 mm slice thickness in the axial plan interleaved by a 0.2 mm gap, covering the entire brain. The fractional extent of brain infarction was calculated from MR images (Pazos et al., 2012), using ImageJ version 1.43u software (NIH). In each slice, the area of each hemisphere was manually outlined, and the size of this area calculated by mean values from ImageJ. The combined area of the 13 consecutive slices was used to calculate the overall volume. A relationship of 0.97 between left hemisphere volumes (LHVs; ipsilateral) and right hemisphere volumes (RHVs; contralateral) in sham animals was used as previously reported (Pazos et al., 2012). In HI animals, the volume of lesion was calculated by subtracting the volume of intact brain tissue in the left hemisphere (ipsilateral) from the theoretical LHV, calculated as RHV × 0.97. The boundary of each lesion was identified by a well defined hyperintense and/or infarcted area. The lesion volume was expressed as a percentage of the theoretical overall brain volume, calculated as RHV + theoretical LHV (= RHV × 0.97). Therefore, the final formula volume of lesion (%) = 49.23 – 100 × intact left volume/(RHV × 1.97).

### Biochemical studies

Levels of oxidized proteins were quantified by Western blot analysis to assess protein carbonylation in ipsilateral temporoparietal cortexes. A detection kit (Millipore Ibérica) was used according to the manufacturer protocol. Oxidized protein levels were quantified via measurement of the optical density, using the NIH ImageJ analysis software. Results were normalized by total protein loading (Red Ponceau staining) and expressed as the OXYBLOT/Red Ponceau ratio.

TNFα Western blot assays were performed with brain samples from ipsilateral temporoparietal cortexes, containing 20 μg of total protein. The protocol used was as previously described (Lafuente et al., 2016). Protein levels (1:1000; rabbit anti-TNFα; Abcam) were quantified using densitometry analysis normalized by β-actin (1:500; Abcam).

### Statistics

The sample size was calculated using G*Power 3.1 (Faul et al., 2007) to provide >80% power to detect a reduction in behavioral outcomes of 45% (range, 40–50%) between the groups. JMP version 6.0 software (SAS Institute) was used for all statistical analyses. Results were compared with a Brown–Forsythe test to confirm the homogeneity of variance between the different treatments, and the distribution of data was assessed using the Shapiro–Wilk test, with the outcome for each dataset described in the figure legends. Normal data are presented by histograms (mean ± SEM), and non-normal histograms are presented as box plots (median with interquartile range).

For normal data, comparisons between the groups were analyzed using one-way ANOVA with Bonferroni–Dunn’s correction as function of group, disease, or treatment. Two-way ANOVA (time vs group) followed by Bonferroni–Dunn’s *post hoc* test was applied to the growth curve (weight vs time). For non-normal data, comparisons were tested with the Kruskal–Wallis test and Dunn’s test for multiple comparisons, as a function of group, disease, or treatment. A *p* value <0.05 was accepted as significant.

## Results

**Figure 1.**
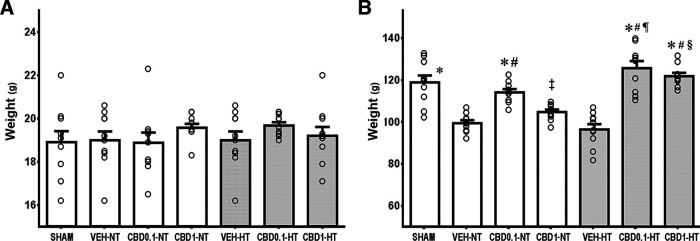
Body weights. ***A***, ***B***, Graph represents the mean body weights at P7 (***A***) and P37 (***B***) of the following three NT and HT groups: HI VEH-treated group (VEH); CBD0.1 group; and HI CBD1 group. The SHAM group was used as a reference. Data are presented by histograms (mean ± SEM) and one-way ANOVA as a function of group, disease or treatment was used. For each group, *N* = 10. JMP version 6.0 was used for all statistical analyses. A *p* value <0.05 was considered to be significant. At P7, all groups demonstrated similar weight (*N* = 10; one-way ANOVA: *F*_(6,63)_ = 0.7142, *p *=* *0.6395). At P37: *p <* *0.05 versus VEH-NT; #*p *<* *0.05 versus VEH-HT; ¶*p *<* *0.05 versus CBD0.1-NT; ‡*p *<* *0.05 versus CBD0.1-HT; §*p *<* *0.05 versus CBD1-NT.

Although an overall mortality rate of 10% (62 of 631) was measured on previous trials or experiments, only two animals in normothermic and hypothermic vehicle-treated groups (one per group) died during the HI insult or during HT treatment (Table 1). Only surviving animals at P37 were included in the study. At P7, all groups demonstrated similar weight (Fig. 1*A*). At P37, growth curves were observed with significant differences (Fig. 1*B*).

Basal rectal temperature was 37.7 ± 0.1°C in all pups (*N* = 10, *F*_(6,63)_ = 0.413, *p *=* *0.868), which was maintained in normothermic animals after the equivalent interval of HT treatment [at 48 h: VEH, 37.5 ± 0.2°C; HI CBD 0.1 mg/kg (CBD0.1)-treated group, 37.6 ± 0.2°C; CBD 1 mg/kg (CBD1)-treated group, 37.5 ± 0.2°C; *N* = 10, *F*_(2,27)_ = 0.133, *p *=* *0.876] and the rewarming phase (at 60 h: VEH group, 37.6 ± 0.2°C; CBD0.1 group, 37.5 ± 0.2°C; CBD1 group, 37.5 ± 0.2°C; *N* = 10, *F*_(2,27)_ = 0.062, *p* = 0.940). In hypothermic groups, the target temperature (32.1 ± 0.2°C) was reached after 18.4 ± 6.5 min. Temperature remained stable throughout HT treatment (48 h) in all groups without differences (VEH group, 32.1 ± 0.1°C; CBD0.1 group, 32.0 ± 0.2°C; CBD1 group, 32.0 ± 0.2°C; *N* = 10, *F*_(2,27)_ = 0.082, *p *=* *0.921). After the rewarming phase (60 h), the hypothermic groups did not demonstrate differences in temperature (VEH group, 37.7 ± 0.2°C; CBD0.1 group, 37.8 ± 0.2°C; CBD1 group, 37.7 ± 0.2°C; *N* = 10, *F*_(2,27)_ = 0.154, *p *=* *0.858). The SHAM group was maintained at constant temperature during all experimental phases (37.1 ± 0.1°C).

### Neurologic functional studies

**Figure 2.**
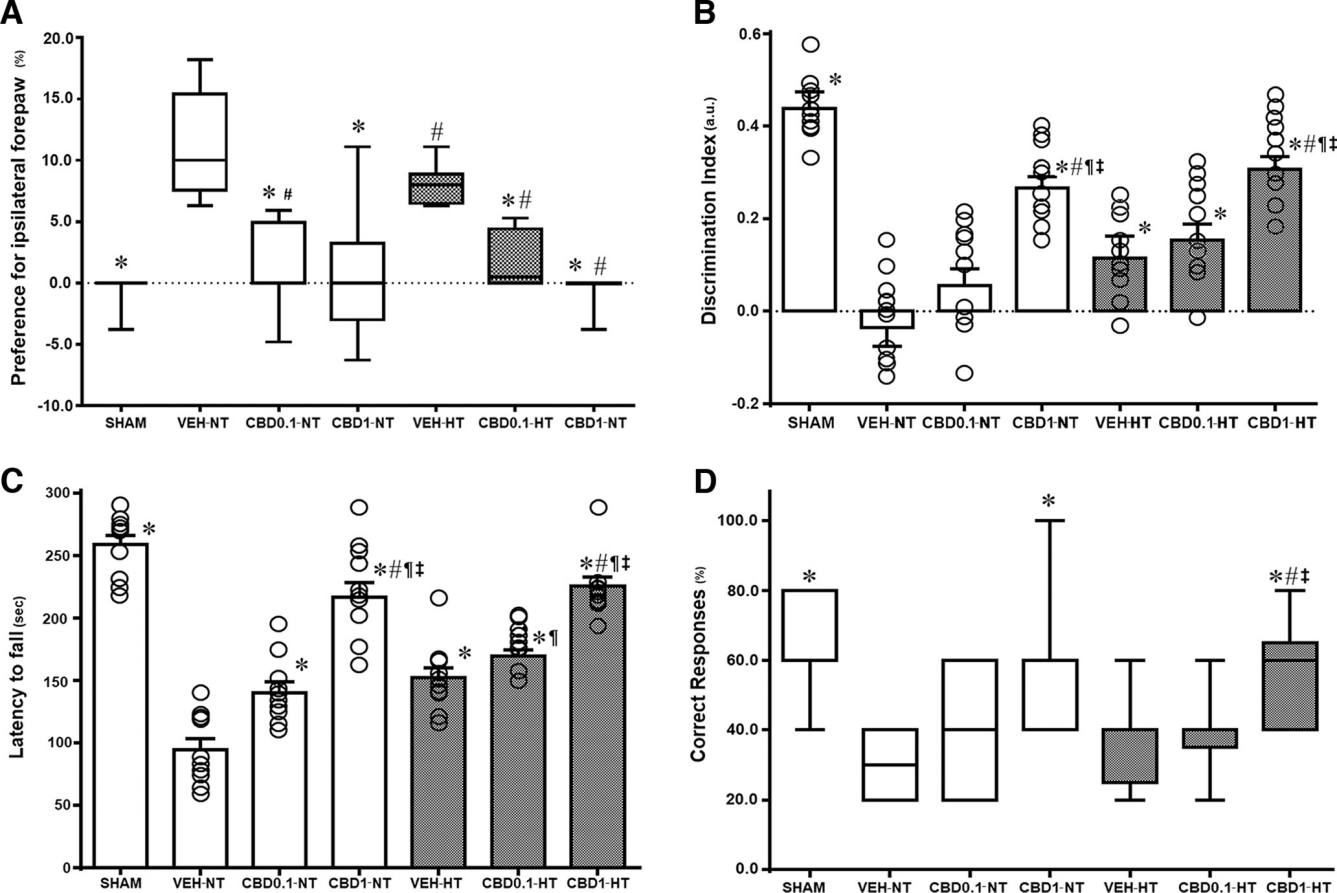
Neurologic functional studies. ***A***–***D***, Cylinder rearing test (***A***), discrimination index of novel object recognition test (***B***), rotarod test (***C***), and spontaneous alternation in a T-maze test (***D***). Graphs represent the mean or median values of the following NT and HT three groups at P37 postnatal age: HI VEH-treated group; HI CBD0.1 group; and HI CBD1 group. The SHAM group was used as a reference. Data in ***A*** and ***D*** are presented as box plots (median with interquartile range), and the Kruskal–Wallis test was used as a function of group, disease, or treatment. Data in ***B*** and ***C*** are presented by histograms (mean ± SEM), and one-way ANOVA was used as a function of group, disease, or treatment. For each group, *N* =10. JMP version 6.0 was used for all statistical analyses. A *p* value <0.05 was considered to be significant. *p <* *0.05 versus VEH-NT; #*p *<* *0.05 versus VEH-HT; ¶*p *<* *0.05 versus CBD0.1-NT; ‡*p *<* *0.05 versus CBD0.1-HT; §*p *<* *0.05 versus CBD1-NT.

The HI insult caused ipsilateral (left) brain damage, resulting in right motor deficit as well as cognitive impairments (Fig. 2*A*). Vehicle-treated animals demonstrated a preference for the ipsilateral forepaw (injured side), a deficit that was not altered by HT treatment (Fig. 2*A*). CBD treatment significantly attenuated this asymmetry at all doses tested to an extent that was also significantly greater than that achieved with HT (Fig. 2*A*).

The HI insult caused a significant reduction in time spent exploring the new objects (Fig. 2*B*), a deficit that was improved by HT treatment alone and 1 mg/kg CBD alone. Furthermore, rats treated with 1 mg/kg CBD spent significantly more time exploring the novel object than did rats treated with HT + VEH (Fig. 2*B*).

Locomotor function was impaired as a consequence of hypoxia-ischemia (Fig. 2*C*), and this was significantly improved in all treatment groups (Fig. 2*C*). Animals treated with CBD 1 mg/kg demonstrated a twofold increase of the latency to fall in relation to vehicle-treated animals, and showed a significantly superior motor coordination compared with HT + VEH-treated rats. In the groups treated with 0.1 mg/kg CBD, the combined use with hypothermia significantly increased the latency to fall compared with normothermic groups receiving a similar dose, but was not significantly different from HT + VEH (Fig. 2*C*).

In relation to the ability to choose the correct response in the T-maze, hypoxia-ischemia resulted in significantly more errors (Fig. 2*D*). Hypothermia alone did not affect performance, but CBD 1 mg/kg treatment significantly improved the percentage of correct responses in both normothermia and hypothermia (Fig. 2*D*).

**Figure 3.**
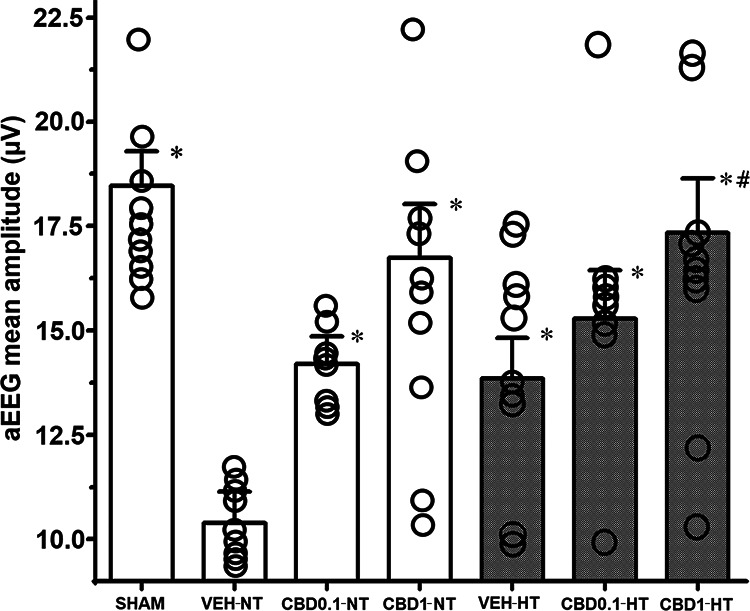
Amplitude-integrated electroencephalography: the graph represents the mean values following NT and HT for three groups at P37: HI VEH-treated group; HI CBD0.1 group; and HI CBD1 group. The SHAM group was used as a reference. Data are presented by histograms (mean ± SEM), and one-way ANOVA was used as a function of group, disease, or treatment. For each group, *N* =10. JMP version 6.0 was used for all statistical analyses. A *p* value <0.05 was considered to be significant. *p <* *0.05 versus VEH-NT; #*p *<* *0.05 versus VEH-HT; ¶*p *<* *0.05 versus CBD0.1-NT; ‡*p *<* *0.05 versus CBD0.1-HT; §*p *<* *0.05 versus CBD1-NT (Extended Data Fig. 3-1).

At P37, the aEEG mean values of juvenile rats showed a significant impairment in the VEH-NT group (Fig. 3). All treated groups showed an improvement of mean aEEG values, and 1 mg/kg CBD combined with HT was significantly superior to hypothermic vehicle-treated rats (Fig. 3). At P37, neither hypoxic-ischemic nor sham animals demonstrated convulsive patterns (seizures) based on the raw EEG traces.

10.1523/ENEURO.0417-22.2023.f3-1 Figure 3-1 ***A***, Algorithm used to obtain the amplitude integrated EEG signal via a step-by-step signal-processing method. ***B***, The following six steps were applied to calculate the compact aEEG tracing: (1) asymmetrical data filtering; (2) absolute value evaluation; (3) envelope detection; (4) tracing compression; (5) segmentation and terminal point extraction; and (6) margin calculation. (7) The median of the amplitude integrated electroencephalography was displayed every successive 20 terminal points. Download Figure 3-1, TIF file.

### Histologic studies

**Figure 4.**
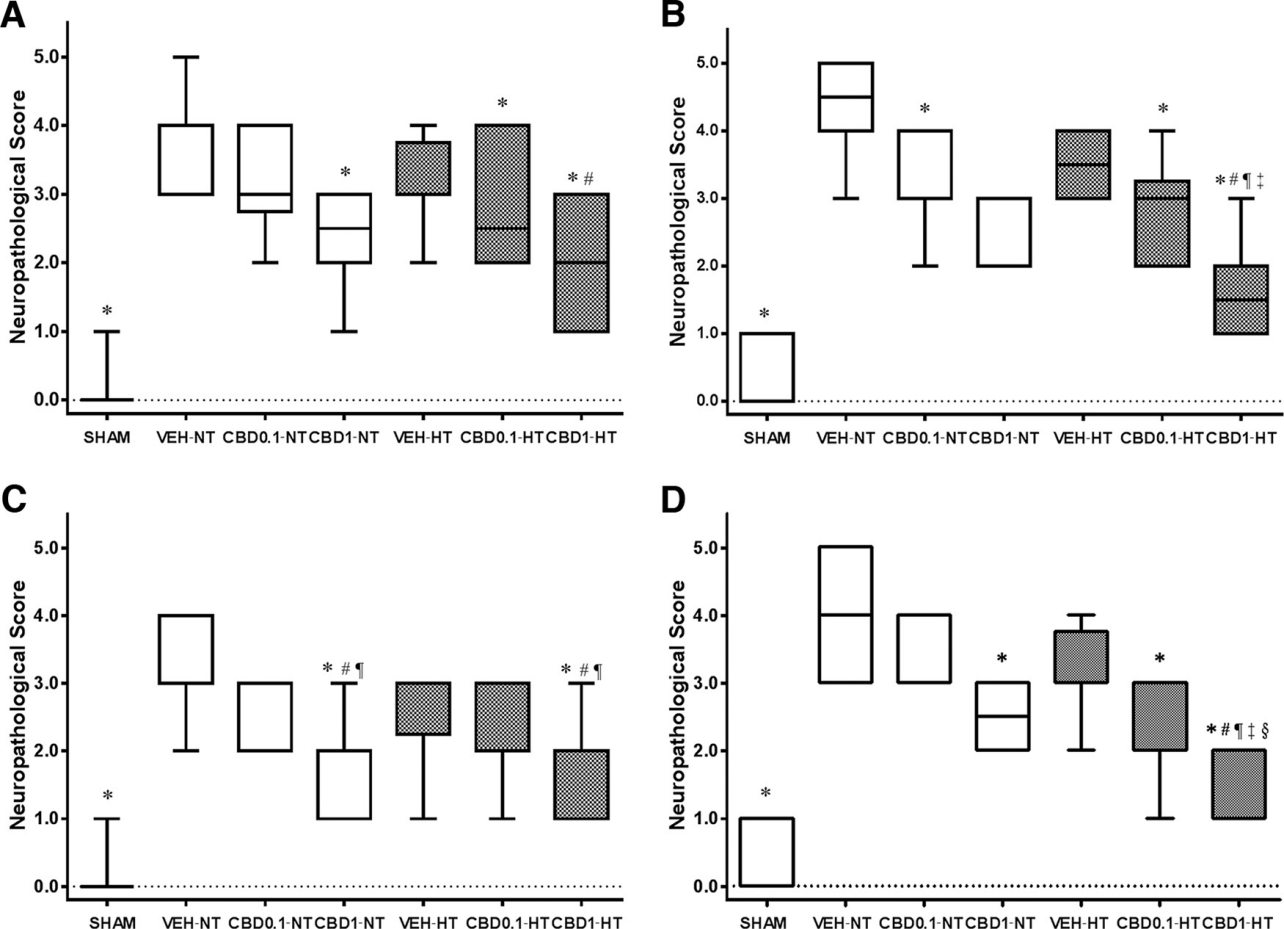
Neuropathological score. ***A–D***, Graph represents the neuropathological scores in the temporoparietal cortex at P14 (***A***) and P37 (***B***), and in the CA1 area of the hippocampus at P14 (***C***) and P37 (***D***), of three groups following NT and HT: HI VEH-treated group, HI CBD0.1 group, and HI CBD1 group. The SHAM group was used as a reference. Data are presented as box plots (median with interquartile range), and Kruskal–Wallis test was used as a function of group, disease, or treatment. For each group, *N* = 5. JMP version 6.0 was used for all statistical analyses. A *p* value <0.05 was considered to be significant. *p <* *0.05 versus VEH-NT; #*p *<* *0.05 versus VEH-HT; ¶*p *<* *0.05 versus CBD0.1-NT; ‡*p* < 0.05 versus CBD0.1-HT; §*p *<* *0.05 versus CBD1-NT.

**Figure 5.**
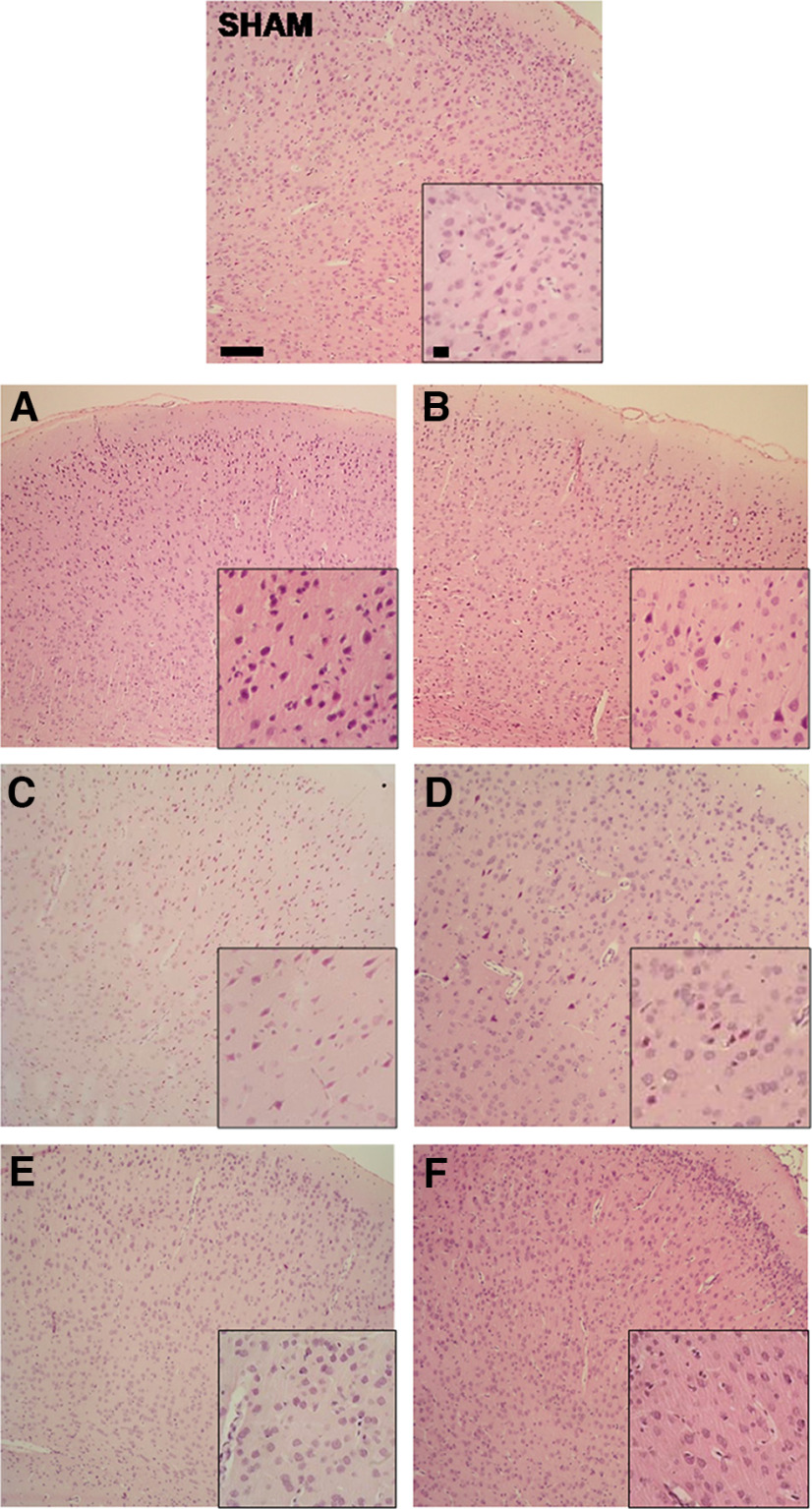
Representative images of ipsilateral temporoparietal cortexes at P37. On top, the SHAM group was used as a reference (neuropathological score, 0). The left column corresponds to NT groups, and the right column corresponds to HT groups, respectively. ***A–F***, HI vehicle groups [VEH-NT (***A***) and VEH-HT (***B***), neuropathological scores 4 and 2, respectively]; HI CBD0.1-NT (***C***) and CBD0.1-HT (***D***) (neuropathological scores 1 and 2, respectively); and CBD1-NT (***E***) and CBD1-HT (***F***) groups (neuropathological scores 1 and 1, respectively). Many neurons in the brains of VEH-treated animals appeared severely damaged. Animals treated with CBD (both 0.1 and 1 mg/kg) reduced the extent of brain damage. Hypothermic VEH group did not significantly display reduction over the normothermic VEH one. However, additive improvement was observed in the combination of 1 mg/kg CBD and HT. Original magnification: 100× (scale bar, 50 μm); insets, 400× (scale bar, 10 μm).

**Figure 6.**
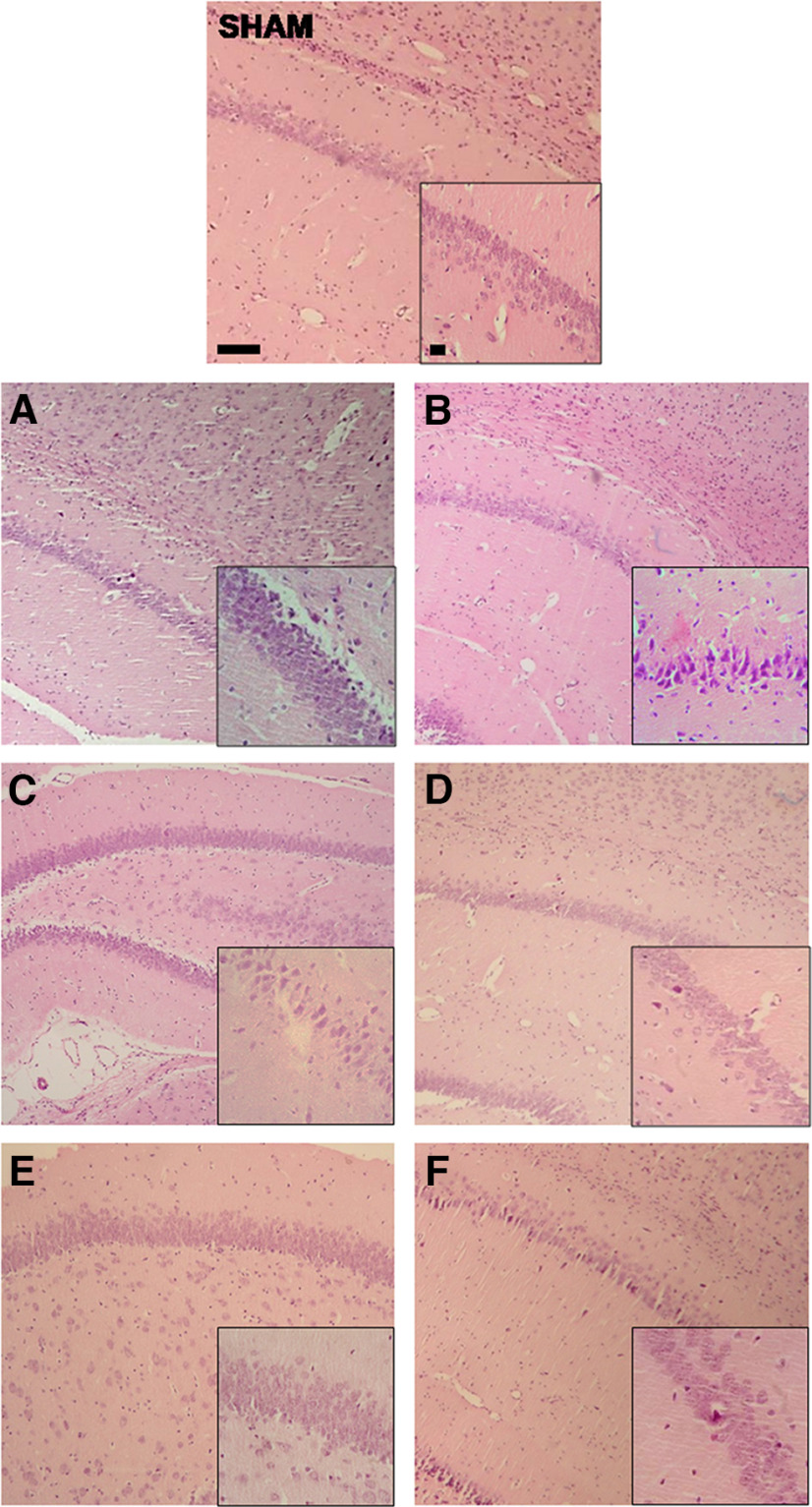
Representative images of the CA1 area of the ipsilateral hippocampus at P37. On top, the SHAM group was used as a reference (neuropathological score, 0). The left column corresponds to NT groups, and the right column corresponds to HT groups, respectively. ***A–F***, HI VEH groups [VEH-NT (***A***) and VEH-HT (***B***) (neuropathological scores 4 and 2, respectively); HI CBD0.1-NT (***C***) and CBD0.1-HT (***D***) groups (neuropathological scores 1 and 1, respectively); and CBD1-NT (***E***) and CBD1-HT (***F***) groups (neuropathological scores 0 and 1, respectively). Neurons in CA1 hippocampal layer of the brains of VEH animals appeared damaged. The damaged area of the hippocampi of CBD-treated animals (both 0.1 and 1 mg/kg concentrations) was reduced. The hypothermic VEH group did not significantly display reduction over the normothermic VEH group. However, additive improvement was observed in the combination of 1 mg/kg CBD and HT. Original magnification: 100× (scale bar, 50 μm); insets, 400× (scale bar, 10 μm).

Hypoxia-ischemia led to an ipsilateral area of infarction with severe damage to the surrounding tissues, including the ipsilateral temporoparietal cortex at P14 (Fig. 4*A*) or P37 (Fig. 4*B*) and the hippocampus at P14 (Fig. 4*C*) or P37 (Fig. 4*D*). Thus, many neurons in the brains of VEH animals appeared severely damaged (neuropathological score exceeding 3 points), both at P14 (Fig. 4*A*,*C*) and P37 (Fig. 4*B*,*D*). Histologic evaluation demonstrated that treatment with CBD reduced the extent of brain damage in almost all groups at P37 in a dose-dependent manner. Overall, there was a mean neuropathological score reduction of 1 point when compared with the brains of the respective VEH groups. Hypothermic vehicle-treated animals did not display benefit over normothermic vehicle animals. Additive effects were observed in the combination of 1 mg/kg CBD with HT at P37 in the hippocampus (Fig. 4*D*). Representative images of ipsilateral temporoparietal cortexes and CA1 hippocampal layers are shown in Figures 5 and 6.

### MRI

**Figure 7.**
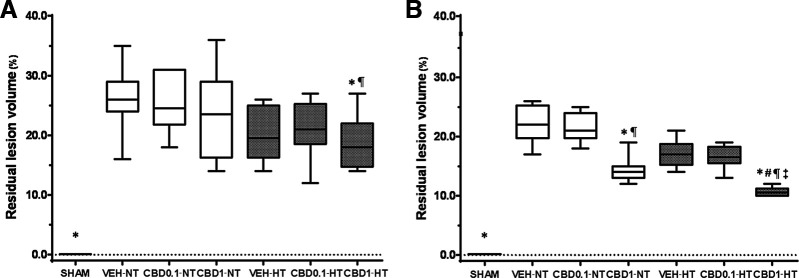
Residual lesion volume. ***A***, ***B***, Graph represents the median values of the following three NT and HT groups at P14 (***A***) and P37 (***B***): HI VEH-treated groups; HI CBD0.1 group, and HI CBD1 group. SHAM group was used as a reference. Data are presented as box plots (median with interquartile range), and the Kruskal–Wallis test was used as a function of group, disease, or treatment. For all groups *N* = 5. JMP version 6.0 was used for all statistical analyses. A *p* value <0.05 was considered to be significant. *p <* *0.05 versus VEH-NT; #*p *<* *0.05 versus VEH-HT; ¶*p *<* *0.05 versus CBD0.1-NT; ‡*p *<* *0.05 versus CBD0.1-HT; §*p *<* *0.05 versus CBD1-NT.

**Figure 8.**
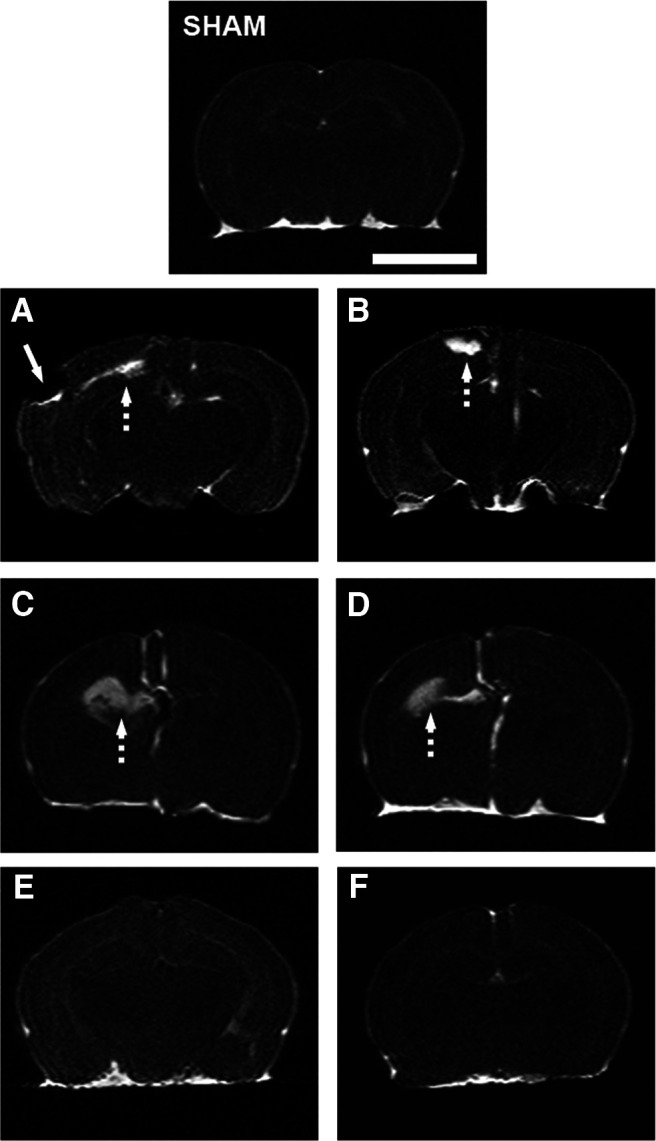
Representative MRI images at P37. On top, the SHAM group was used as a reference. The left column corresponds to NT groups, and the right column corresponds to HT groups, respectively. ***A–F***, HI VEH groups [VEH-NT (***A***) and VEH-HT (***B***)]; HI CBD0.1-NT (***C***) and CBD0.1-HT (***D***) groups; and CBD1-NT (***E***) and CBD1-HT (***F***) groups. In the vehicle-treated normothermic animals (***A***), a great defect can be observed in the left hemisphere (white arrow) as well as an infarcted hyperintense area located near the hippocampus (dashed arrow). Infarcted hyperintense areas (dashed arrows) are also shown in ***B***–***D***. All images are shown at the same scale. White scale bar, 5 mm.

Rats exposed to hypoxia-ischemia had detectable lesion volumes of ∼25% at P14 and P37. At P14, the only treatment that significantly reduced the lesion volume was CBD 1 mg/kg combined with HT (Fig. 7*A*). At P37, animals treated with CBD 1 mg/kg significantly decreased the volume of lesion. Combination of CBD 1 mg/kg with HT resulted in significantly superior effects compared with HT alone (Fig. 7*B*). Representative images of all groups at this developmental age are included in Figure 8.

### H^+^-MRS

Rats exposed to hypoxia-ischemia showed increased Lac/NAA ratios (metabolic dysfunction), which were evident at P14 and P37 (Table 2). At P14, this effect was not altered by treatment with CBD or HT, but CBD 1 mg/kg + HT had significantly lowered Lac/NAA ratios compared with HT alone (Table 2). At P37, treatment with HT or CBD 1 mg/kg significantly reduced the Lac/NAA ratio. (Table 2).

**Table 2:** Brain metabolites measured by proton magnetic resonance spectroscopy of the following three NT and HT groups at P14 and P37 postnatal age: HI VEH, HI CBD0.1, and HI CBD1

	Lac/NAA		Glu/NAA		NAA/Cho		Lac/Cr	
	NT	HT	NT	HT	NT	HT	NT	HT
P14								
SHAM	1.6 (1.9, 1.4)*	1.0 (1.4, 0.8)*	5.3 (5.7, 5.0)*	1.3 (1.5, 1.1)*				
VEH	2.7 (3.4, 2.2)	2.9 (3.0, 2.7)	1.4 (1.6, 1.3)	1.5 (1.7, 1.4)	2.0 (2.2, 1.9)	4.2 (4.4, 3.9)*	1.9 (2.0, 1.7)	2.1 (2.4, 2.0)*
CBD0.1	2.4 (2.6, 2.3)	2.5 (3.0, 2.3)	1.4 (1.6, 1.2)	1.3 (1.4, 1.1)*#¶§	5.3 (5.8, 5.0)*#	5.4 (5.9, 5.1)*#	1.8 (2.1, 1.6)#	2.0 (2.3, 1.7)
CBD1	2.8 (3.0, 2.6)	2.0 (2.3, 1.7)#	1.6 (1.7, 1.3)	1.3 (1.4, 1.1)*#¶§	4.8 (5.7, 4.2)*#	4.2 (4.4, 3.8)*¶‡	1.5 (1.8, 1.2)#‡	1.5 (1.7, 1.4)#
P37								
SHAM	1.9 (2.2, 1.8)*	1.2 (1.3, 1.0)*	8.5 (8.9, 8.2)*	2.4 (2.5, 2.2)*				
VEH	3.4 (3.6, 2.7)	1.8 (2.0, 1.6)*	1.7 (1.9, 1.6)	1.0 (1.1, 0.9)*	2.8 (3.5, 2.4)	8.3 (8.4, 8.1)*	2.8 (3.0, 2.6)	2.3 (2.7, 2.2)*
CBD0.1	2.9 (3.1, 2.7)#	1.9 (2.2, 1.8)*¶	1.2 (1.2, 1.1)*	1.0 (1.2, 0.9)*	3.8 (4.4, 3.1)#	8.5 (9.0, 8.3)*¶	2.4 (2.6, 2.2)*	2.5 (2.7, 2.2)*
CBD1	2.0 (2.8, 1.5)*¶	1.8 (2.0, 1.6)*¶	1.2 (1.5, 1.0)*#	1.0 (1.1, 0.8)*	4.4 (5.1, 3.7)#‡	9.9 (10.0, 9.7)*#§¶	2.2 (2.6, 2.1)*	2.3 (2.5, 2.1)*

Values are the median (interquartile range). The Kruskal–Wallis test was used as a function of group, disease or treatment. For each group, *N* = 5. JMP version 6.0 was used for all statistical analyses. The SHAM group was used as a reference.

*p <* *0.05 versus VEH-NT; # *p *<* *0.05 versus VEH-HT; ¶ *p *<* *0.05 versus CBD0.1-NT; ‡ *p *<* *0.05 versus CBD0.1-HT; § *p *<* *0.05 versus CBD1-NT.

Exposure to HI injury increased the Glu/NAA ratio (excitotoxicity) at P14 and P37. At P14, the HT and CBD treatments alone did not alter the Glu/NAA ratio, but the combination of CBD and HT reduced this ratio at both doses, indicating additive effects (Table 2). At P37, all treatment groups demonstrated significant reductions in excitotoxicity compared with vehicle (Table 2).

Also, we observed that hypoxia-ischemia reduced the NAA/Cho ratio (neural damage) at both P14 and P37 (Table 2). At P14, this effect was significantly ameliorated in all treatment groups. Combination of HT with CBD 0.1 mg/kg resulted in significantly superior effects to HT alone (Table 2). At P37, the NAA/Cho ratio was significantly increased with CBD 0.1 mg/kg and CBD 1 mg/kg, but to a lesser extent than HT alone. When HT was combined with CBD 1 mg/kg there was an additive effect (Table 2).

The Lac/Cr ratio (mitochondrial impairment) was increased in HI animals at P14 and P37 (Table 2). At P14, this effect was not significantly altered in CBD-treated groups. Indeed, treatment with hypothermia resulted in an exacerbation of this, an effect that was significantly reduced when given in combination with CBD 1 mg/kg (Table 2). At P37, all treatment groups significantly reduced the Lac/Cr ratio (Table 2).

### Biochemical studies

**Figure 9.**
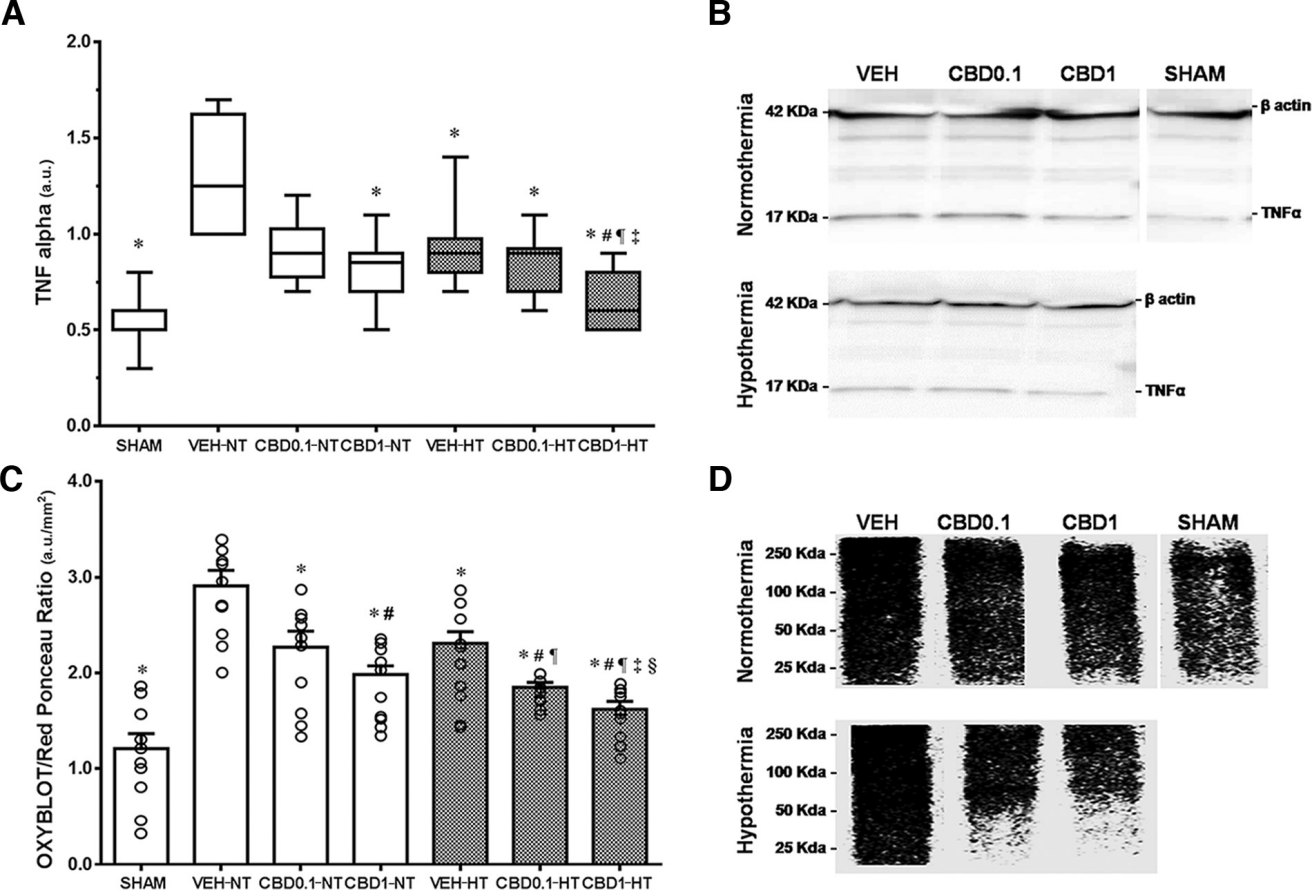
Western Blot. ***A***, ***B***, Graphs represent the median values (***A***) and an illustrative image of TNFα (***B***) of the following three NT and HT groups at P37: HI VEH-treated groups, HI CBD0.1 group, and HI CBD1 group. ***C***, ***D***, Mean values of protein carbonyl formation (Oxyblot; ***C***) and an illustrative image of the same groups are also included (***D***). The SHAM group was used as a reference. Data in ***A*** are presented as box plots (median with interquartile range), and the Kruskal–Wallis test was used as a function of group, disease, or treatment. Data in ***C*** are presented by histograms (mean ± SEM), and one-way ANOVA was used as a function of group, disease, or treatment. For all groups, *N* = 10. JMP version 6.0 was used for all statistical analyses. A *p* value <0.05 was considered to be significant. *p <* *0.05 versus VEH-NT; #*p *<* *0.05 versus VEH-HT; ¶*p *<* *0.05 versus CBD0.1-NT; ‡*p *<* *0.05 versus CBD0.1-HT; §*p *<* *0.05 versus CBD1-NT.

Exposure to hypoxia-ischemia significantly elevated TNFα levels (Fig. 9*A*). HT and CBD 1 mg/kg treatment led to a reduction of TNFα content in the ipsilateral temporoparietal cortex compared with the normothermic-vehicle group (Fig. 9*A*). Rats treated with CBD 1 mg/kg in combination with HT had significantly lower TNFα levels than those treated with HT alone (Fig. 9*B*).

An increased mean value of OXYBLOT/Red Ponceau ratio in the analyzed proteins from the temporoparietal cortex was observed following hypoxia-ischemia (Fig. 9*C*), which was reduced in all treatment groups (Fig. 9*C*), indicating a reduction of protein carbonylation. Furthermore, treatment with CBD 1 mg/kg was significantly superior to that with HT alone, and the combination therapy of CBD with HT reduced oxidative stress to an extent that was significantly superior to treatment with HT alone (Fig. 9*D*).

### Pharmacokinetic studies

The CBD concentrations in brain tissue and plasma, the maximum *t* value (*t*_max_), maximum concentration (C_max_), and the area under the curve (AUC), in normothermic and hypothermic conditions, at 0.5, 1, 6, 12, 24, and 36 h, are shown in Table 3.

**Table 3:** Pharmacokinetic of CBD for plasma and brain samples obtained from P7 rats that have been exposed to HJI and administered CBD1 30 min post-HI insult, in either NT or HT environment

	Time after treatment						*t*_max_(h)	C_max_(ng/ml)	AUC(ng/ml.min-1)
	0.5 h(ng/ml)	1 h(ng/ml)	6 h(ng/ml)	12 h(ng/ml)	24 h(ng/ml)	36 h(ng/ml)			
CBD dose at 0.1 mg/kg									
Plasma									
NT	12 (13, 12)	8 (10, 7)	n.d.	n.d.	n.d.	n.d.	0.5	12 (13, 12)	29 (33, 27)
HT	8 (13, 4)	11 (12, 10)	n.d.	n.d.	n.d.	n.d.	1.0	11 (12, 10)	33 (36, 31)
Brain									
NT	n.d.	n.d.	n.d.	n.d.	n.d.	n.d.	n.d.	n.d.	n.d.
HT	n.d.	n.d.	n.d.	n.d.	n.d.	n.d.	n.d.	n.d.	n.d.
CBD dose at 1 mg/kg									
Plasma									
NT	102 (111, 98)	52 (59, 41)	14 (16, 12)	5 (6, 4)	n.d.	n.d.	0.5	102 (111, 98)	292 (347, 242)
HT	328 (436, 296)*	192 (212, 161)*	37 (50, 28)*	6 (9, 5)	n.d.	n.d.	0.5	328 (436, 296)*	1010 (1680, 896)*
Brain									
NT	123 (164, 88)	72 (80, 60)	0 (4, 0)	0 (4, 0)	n.d.	n.d.	0.5	123 (164, 88)	265 (368, 206)
HT	72 (81, 64)*	126 (130, 110)	8 (17, 0)	n.d.	n.d.	n.d.	1.0	126 (130, 110)	401 (440, 363)

Values are the median (interquartile range). Samples were taken at 0.5, 1, 6, 12, 24, or 36 h after the drug administration. *N* = 5 per each time interval. n.d., Not detectable. JMP version 6.0 was used for all statistical analyses.

*p <* *0.05 normothermic versus hypothermic groups by the Mann–Whitney test.

At a CBD dose of 0.1 mg/kg, CBD was not detected in brain samples. In plasma samples, CBD C_max_ in normothermic hypoxic-ischemic animals was 12 ng/ml at 0.5 h (*t*_max_), but in hypothermic hypoxic-ischemic animals it was 11 ng/ml at 1 h (*t*_max_).

At a CBD dose of 1 mg/kg in plasma samples, CBD C_max_ values in normothermic and hypothermic hypoxic-ischemic animals were 102 and 328 ng/ml, respectively, at the same *t*_max_ (0.5 h). However, in brain samples, the CBD C_max_ in normothermic hypoxic-ischemic animals was 123 ng/ml at 0.5 h (*t*_max_) but in hypothermic hypoxic-ischemic animals it was 126 ng/ml at 1 h (*t*_max_). Brain and plasma levels were significantly higher in HT-treated compared with NT-treated rats following administration of 1 mg/kg CBD.

Also, it was demonstrated that after 12 and 24 h of the last CBD dose, no residual levels in brain or plasma were detected at 0.1 and 1.0 mg/kg CBD, respectively.

## Discussion

We found that hypothermia or CBD treatments improved brain activity, cognitive, and motor functions in HI animals, although CBD 1 mg/kg was superior to HT treatment in rotarod and CRT tests. Rats treated with 1 mg/kg CBD + HT were superior to those treated with HT alone in all parameters, with additivity present for excitotoxicity amelioration at P14, and reductions in neural damage and neuropathological score at P37. CBD administration after neonatal hypoxia-ischemia recovered the sensorimotor performance tests to sham-like levels at P37. This is important since well recognized neuroprotective strategies, such as hypothermia, improve performance several weeks after hypoxia-ischemia with behaviors not returning to control levels (Lee et al., 2010). Hypothermia treatments in rodents are limited to a duration of up to 6 h (Thoresen et al., 1996; Mueller-Burke et al., 2008; Rocha-Ferreira et al., 2018), with positive results. Using a treatment duration of 48 h, the same efficacy of hypothermia was observed, but with reduced efficacy compared with the combination of hypothermia and CBD, or CBD alone.

Hypoxia-ischemia led to memory deficits in both sensorimotor tests, as indicated by a 30% reduction in preference for new objects, as well as a 10% increase of preference for ipsilateral forepaw in the VEH group, which are similar to other studies (Simola et al., 2008; Lee et al., 2010; Pazos et al., 2012; Duran-Carabali et al., 2019). This deficit may be explained by extensive damage in the hippocampus and cerebral cortex. The hippocampus and its connections to the cerebral cortex are responsible for recognition memory (Dere et al., 2007; Broadbent et al., 2010). Impairments in reference and working memory evaluated in the T-maze performance have been demonstrated in previous studies following neonatal hypoxia-ischemia (Simola et al., 2008; Pazos et al., 2012). We observed similar results, showing that 1 mg/kg CBD and hypothermia improved the percentage of correct responses. CBD improved memory deficits (preference for ipsilateral forepaw and for new objects) caused by hypoxia-ischemia, reducing hippocampal and cortical damage. Few studies have explored the effect of long-term neuroprotective strategies on memory deficits in this neuropathology (Lee et al., 2010; Pazos et al., 2012). Hypothermia treatment has been shown to reduce, but not prevent, spatial memory deficits after hypoxia-ischemia in newborn rats (Wagner et al., 2002), demonstrating only a 5% reduction of preference for ipsilateral forepaw in hypoxic-ischemic P37 rats (Lee et al., 2010).

Our results demonstrate an improvement in neurocognitive and neuromotor functions, preserving brain activity, when rats were administered both CBD and hypothermia. Hypoxia-ischemia in newborn rats resulted in long-lasting brain damage. The ipsilateral brain hemisphere volume was reduced by 40%, similar to results in other studies (Wagner et al., 2002; Lee et al., 2010). The tissue surrounding the cortical infarcted area appeared damaged, which is typical in this experimental model (Wagner et al., 2002; Hobbs et al., 2008; Rocha-Ferreira et al., 2018). However, CBD reduced histologic brain damage, and neuropathological scores were lower in both hippocampus and cerebral cortex compared with VEH-treated animals. Furthermore, CBD promoted functional recovery since neurobehavioral measures approached normal levels. Existing studies described a partial recovery of brain function after CBD administration in HI-injured piglets at short term (Alvarez et al., 2008; Lafuente et al., 2011, 2016; Pazos et al., 2013; Barata et al., 2019). This study shows, for the first time, the long-term efficacy of CBD in HIE neonatal and juvenile rats. The determination of the aEEG in P7 neonatal rat is complicated because of their small size and the motor activity of the unrestrained animals interfering with the recording of the raw EEG signal (Tucker et al., 2009; Zayachkivsky et al., 2013). Despite this, we quantified brain activity in unconscious P37 juvenile animals under inhaled anesthesia. Thus, in addition to having a more suitable size of juvenile animals for measurements and correct recording of the EEG signal, we avoided signal disturbance by movement artifacts. Even under these conditions, recovery of brain activity was evident in animals receiving either hypothermia or CBD, but a combination of both showed a greater aEEG improvement.

At P14, the brain damage volume assessed by MRI was similar in VEH and CBD groups, while it was reduced in CBD-treated animals at P37. This difference between P14 and P37 is likely because hyperintense areas in T2WI shortly after hypoxia-ischemia correspond to areas of brain edema, including necrotic tissue and injured but recoverable tissue, the so-called “penumbral” area (Fernández-López et al., 2007). CBD exerted a protective effect on brain tissue surrounding the cortical infarcted area at P14, showed by histologic and Western blot results. These findings suggest that CBD administration recovers the penumbral area, preventing necrosis and reducing the damage in the neonatal HI model, as previously shown in other neonatal models (Lafuente et al., 2011; 2016; Ceprián et al., 2017; Barata et al., 2019). The final reductions of brain damage volume and neuropathological score by CBD were greater than hypothermia alone.

Excitotoxicity, inflammation, and oxidative stress damage the immature brain (Johnston et al., 2011; Drury et al., 2014; Juul and Ferriero, 2014). The high concentration and activity of glutamate receptors aggravates the deleterious effects of excitatory amino acids in immature brains (Johnston et al., 2011; Drury et al., 2014; Juul and Ferriero, 2014). The Glu/NAA ratio has been observed to be closely related to excitotoxicity (Dang and Wang, 2020). Antioxidants and excess of pro-oxidant substances determine the susceptibility of the immature brain to oxidative stress (Johnston et al., 2011). HI-induced increases in oxidative stress can be determined by protein carbonylation, leading to brain functional loss after ischemia/reperfusion (Oikawa et al., 2009) and detectable early after hypoxia-ischemia (Mueller-Burke et al., 2008; Pazos et al., 2013). Inflammation in acute brain damage and its long-lasting impact on the immature brain after HI insults plays a crucial role in neuropathophysiology (Johnston et al., 2011; Drury et al., 2014; Juul and Ferriero, 2014; DeFrancesco-Lisowitz et al., 2015). TNFα increase after hypoxia-ischemia occurs as early as 1 h after HI insult, correlating with the extent of tissue injury and clinical outcomes (DeFrancesco-Lisowitz et al., 2015).

Our results show that CBD treatment is associated with reductions in HI damage from excitotoxicity (blocking the HI-induced increase in Glu/NAA ratio), oxidative stress (preventing the HI-induced increase in carbonylation protein), and inflammation (reducing the HI-induced increase in TNFα production). CBD is a strong antioxidant because of its molecular properties (Zou and Kumar, 2018) and also because it modulates inducible nitric oxide synthase expression, a major source of free radicals after hypoxia-ischemia (Johnston et al., 2011; Drury et al., 2014; Juul and Ferriero, 2014), as demonstrated in forebrain slices of newborn mice after oxygen-glucose deprivation (Castillo et al., 2010). Furthermore, it reduces the release of cytokines (IL-1, IL-6, and TNFα) after an HI insult, as demonstrated in newborn rodents *ex vivo* (Castillo et al., 2010) and *in vivo* (Pazos et al., 2012).

The combination of hypothermia and CBD led to a better modulation of excitotoxicity and inflammation than that obtained with either treatment alone; suggesting that the mechanisms by which CBD and hypothermia modulate those factors are different. Thus, CBD might be a suitable partner for hypothermia in excitotoxic and inflammatory cascades. Additional studies are needed to clarify the role of other elements that influence these cascades, such us, membrane pump function, glutamate release, inflammatory cytokines, nitric oxide activation, or free radicals. CBD showed a very similar profile to hypothermia regarding its effects on excitotoxicity, inflammation, and oxidative stress. Some cannabinoid drugs, such as tetrahydrocannabinol or cannabinol, interfere with thermoregulation and could augment the hypothermia treatment. However, CBD did not demonstrate an effect on body temperature in rats at intraperitoneal doses of 10 or 30 mg/kg (Taffe et al., 2015; Javadi-Paydar et al., 2019). Only the vapor inhalation route has produced hypothermia in rats exposed to vapor-generated CBD (Javadi-Paydar et al., 2019). The current study demonstrates that CBD has no long-term hypothermic effects. PK plasma data showed that HT-treated rats had a higher area under curve values than NT treated when coadministered CBD 1 mg/kg. This altered PK profile could be a consequence of kinetics retardation that occurs in HT animals, and the higher levels could be explained by the fact that genuine *t*_max_ in NT animals occurred before the first reading (30 min). It has been previously reported that healthy rat pups showed a CBD C_max_ of 13.9 ± 2.6 ng/g at 3 h (*t*_max_) in brain tissue (Pazos et al., 2012). We determined the PK curve of CBD in HI rat pups with normothermia or hypothermia. Comparison of PK values between these studies is difficult based on the different formulations, sampling times, and administration routes.

The neuroprotective efficacy of hypothermia is based on the decrease and/or slowdown of neuronal metabolism, with the consequent decrease in oxygen consumption rates and energy needs. Metabolic changes associated with hypothermia include decreased brain glucose levels, decreased lactate release, and increased plasma levels of glycerol, free fatty acids, and ketoacids (Schaller and Graf, 2003). Thus, hypothermia induces beneficial metabolic changes for the maintenance of neuronal ATP, favoring brain homeostasis (Kuffler, 2010). Although the mechanism of action of hypothermia is still largely unknown, it is believed to involve multiple targets and to participate in the suppression of different inflammatory pathways, inhibiting proinflammatory cytokines, and promoting anti-inflammatory cytokines (Okazaki et al., 2023), and would reduce excitotoxicity (Drury et al., 2014) and programmed cell death associated with the caspase family (Shamas-Din et al., 2011). CBD possesses affinity and functional activity at many molecular targets (Ibeas Bih et al., 2015), and thus the mechanisms by which CBD exerts its neuroprotective effects after neuronal damage are not fully understood. CBD prevents excitotoxicity caused by excessive accumulation of glutamate, microglial activation, neurovascular reactivity, inflammatory response, and cell death (Johnston et al., 2011; Juul and Ferriero, 2014; Martínez-Orgado et al., 2021). In addition, CBD can reduce neuronal excitability and transmission by modulating intracellular Ca^++^ levels through different membrane receptors (Zou and Kumar, 2018). Although both therapies modulate their neuroprotective functions on some common pathways, the retardation of metabolic pathways induced by hypothermia could partially explain the delayed effect exerted by CBD. In fact, the response is very similar when comparing the results of the groups that received only CBD and that of the combined hypothermia plus CBD therapy.

In conclusion, CBD administration after HI injury to newborn rats leads to long-lasting neuroprotective effects. In contrast to other studies, CBD was more effective in recovering functional parameters than restoring histologic markers. CBD and hypothermia act on the same processes related to HI brain damage. The two therapies in combination do not compete against each other in modulating these processes, but increase the neuroprotective effects, resulting in greater overall benefit in some parameters. Furthermore, the combination of CBD with hypothermia often resulted in a significantly superior profile compared with hypothermia alone, being a promising observation for the clinic. Our results justify the interest in CBD for developing a translational strategy combining treatments with hypothermia to improve outcome in asphyxiated infants.

## Synthesis

Reviewing Editor: Benjamin Deneen, Baylor College of Medicine Decisions are customarily a result of the Reviewing Editor and the peer reviewers coming together and discussing their recommendations until a consensus is reached. When revisions are invited, a fact-based synthesis statement explaining their decision and outlining what is needed to prepare a revision will be listed below. The following reviewer(s) agreed to reveal their identity: Donna Ferriero, Javier Fernandez-Ruiz. Note: If this manuscript was transferred from JNeurosci and a decision was made to accept the manuscript without peer review, a brief statement to this effect will instead be what is listed below.

The reviewers felt this manuscript is of great interest to the field and is a generally well-conducted study. The reviewers highlighted a few areas that need clarification and some technical technical details that also need further elaboration. Please see the comments below, as they will help further sculpt the main message of this interesting manuscript.

Reviewer 1

this is an interesting study using annabidiol and hypothermia for neonatal HIE in the rat Vannucci model. The introduction is well written and sets up the premise well The results are interesting, strong and promising. However, there are a number of methodological issues that need clarification.

In the abstract, the treatment with cannabidiol suggest that it was given from 7- 37 days. Please correct this.

P7 rats were used. The new standard for term HIE is P10. Please make a comment in the methods in this regard. The treatment with 10%O2 is a rather mild insult compared to what is reported in the literature. Please comment.How many pups were resuscitated37.5 oC is warm for normothermia and may have skewed the results.Were the pups returned to the dam at some point? If so, when?Please use term “forepaw” instead of “arm” Why were animals partially food restricted? Did they become hypoglycemic?In figures 4 and 5, how would those brains be scored?Were the brains for MRI different from those for histology?

For animals that were resuscitated, did this increase injury?

In the results, Line 320, p 12, it states that AT 7 days all groups demonstrated similar weight. Was this really 14days? Please describe completely the mortality in this study.

In line 354, p 14. It is stated that CBD and HT reduced latecy to fall-what is this in comparison to?

In figure 1, 10% is not a large amount for paw reference, please comment.

The discussion is well written. There should be some mention of the difference between P7 and P10 which is now standard for term HIE.

Figure 7 is difficult to visualize.IS F severe?

Reviewer 2

This study investigated at the preclinical level the benefits of the combination of hypothermia and cannabidiol in the treatment of newborn hypoxic-ischemic encephalopathy. Hypothermia is the reference clinical treatment for this pathology, but it does not work in all cases and its effects are modest, whereas the treatment with cannabidiol has been found to be active using preclinical models. Their combination appears an objective of interest which has been already investigated with success in some experimental models in piglets, so that the present study comes to add more data and confirm the relevance of this combination, then supporting the interest to urgently move this therapeutic strategy towards the clinic. Despite the interest of the data provided here, the study and the manuscript could be improved in the following aspects:

1. A missing information in this manuscript is the identification of the targets (e.g. receptors) and signaling pathways that mediate the positive effects of cannabidiol in newborn hypoxic-ischemic encephalopathy, in particular those that facilitate its combination with hypothermia and that explain the additive effect found. This additional information is extremely relevant to facilitate a further progress in the clinical validation of this combination. I would recommend that authors may carry out some additional experiments to identify these mechanisms, or, at least, that the question may be addressed in the Discussion.

2. The experimental model used in this study is based on brain damage occurring only in the ipsilateral side (pathological condition) with the contralateral side reflecting the “healthy” condition and serving as a control for the same animal. This is a clear advantage that is not used in all measurements, for example in the histological, H+-MRS and biochemical data. Following the authors” description, it appears that these data were obtained in cortical and hippocampal tissues that included both the ipsilateral and contralateral sides, right? In my opinion, it would be necessary to measure these data separately in the ipsilateral and the contralateral sides, and they should be presented as different bars or as % of the ipsilateral versus the contralateral.

3. The question of gender should be considered as an important objective in this study. Why authors are assuming that the effects of the combined treatment are similar in males and females? As they have cohorts of at least 4 different subjects for each measurement, I think that it would be interesting to present the data separating males and females too.

4. The weight data should be presented better in graphic form.

5. Results section: The meaning of the different parameters obtained in the H+-MRS analysis should be briefly described the first time that each parameter is mentioned.

6. The description of the most important results in the Abstract (lines 56-61) is confuse and should be rewritten.

7. The statistical methods used for each group of data must be indicated in the different legends to figures.
