# Supplementary figures and images for: Effects of Cannabidiol, Hypothermia, and Their Combination in Newborn Rats with Hypoxic-Ischemic Encephalopathy
Source: eNeuro. 2023 May 4;10(5):ENEURO.0417-22.2023. doi: 10.1523/ENEURO.0417-22.2023 (PMC10166126; doi:10.1523/ENEURO.0417-22.2023)

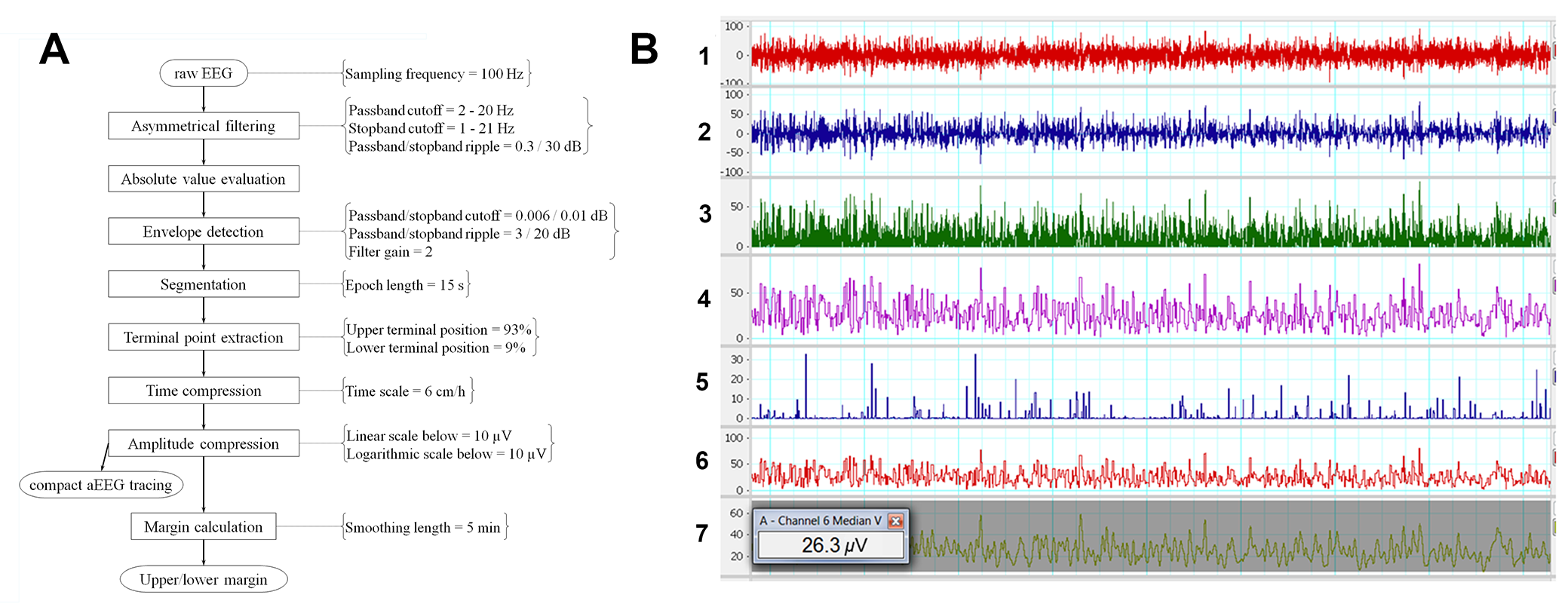

Supplement: Figure 3-1 — A, Algorithm used to obtain the amplitude integrated EEG signal via a step-by-step signal-processing method. B, The following six steps were applied to calculate the compact aEEG tracing: (1) asymmetrical data filtering; (2) absolute value evaluation; (3) envelope detection; (4) tracing compression; (5) segmentation and terminal point extraction; and (6) margin calculation. (7) The median of the amplitude integrated electroencephalography was displayed every successive 20 terminal points. Download Figure 3-1, TIF file. [file enu-eN-NWR-0417-22-s01.tif]
